# Do Maternal Factors and Milk Expression Patterns Affect the Composition of Donor Human Milk?

**DOI:** 10.3390/nu13072425

**Published:** 2021-07-15

**Authors:** Agnieszka Bzikowska-Jura, Natalia Machaj, Piotr Sobieraj, Olga Barbarska, Gabriela Olędzka, Aleksandra Wesolowska

**Affiliations:** 1 Department of Clinical Dietetics, Faculty of Health Sciences, Medical University of Warsaw, E Ciolka Str. 27, 01445 Warsaw, Poland; agnieszka.bzikowska@wum.edu.pl; 2 Department of Medical Biology, Faculty of Health Sciences, Medical University of Warsaw, Litewska Str. 14/16, 00575 Warsaw, Poland; nataliaa146@interia.pl (N.M.); olga.barbarska@wum.edu.pl (O.B.); gabriela.oledzka@wum.edu.pl (G.O.); 3 Department of Internal Medicine, Hypertension and Vascular Diseases, Faculty of Medicine, Medical University of Warsaw, Banacha Str. 1a, 02097 Warsaw, Poland; piotr.sobieraj@wum.edu.pl; 4 Laboratory of Human Milk and Lactation Research, Regional Human Milk Bank in Holy Family Hospital, Department of Medical Biology, Faculty of Health Science, Medical University of Warsaw, Litewska Str. 14/16, 00575 Warsaw, Poland

**Keywords:** human milk composition, breastfeeding, milk banking, donor milk

## Abstract

A primary role of Human Milk Banks (HMBs) is to provide human milk (HM) for preterm infants and to support the mothers of these infants as they establish their own milk supply. A better understanding of the variation in the energy and macronutrients contents of donor human milk (DHM) potentiates targeted nutrition for preterm babies. Therefore, the aim of this study was to assess the variability of energy and macronutrients content in DHM and to investigate the impact of maternal factors and feeding practices on the nutritional value of DHM. The study involved 49 donors registered in the HMB in the Holy Family Hospital in Warsaw, Poland. Samples from each donor were pooled within a maximum of two weeks. The composition of DHM, including energy content, protein, fat, and carbohydrate concentrations, was analyzed using the Miris Human Milk Analyzer. The analyses were performed before the pasteurization process. The mean time of milk donation to HMB was 13.2 ± 6.0 weeks. There were no significant differences in energy and macronutrients contents of DHM in the beginning and at the end of milk donation to HMB, however, HM fat concentration was positively correlated with afternoon feedings (r = 0.289, *p* = 0.044). The method of feeding (breastfeeding vs. feeding only expressed milk) also did not impact the nutritional value of DHM. Future research for the DHM should include a further cross-sectional observational study with the collection of detailed donor information and characteristics of milk expression and feeding practices to further evaluate the pooling processes and the effect on DHM composition.

## 1. Introduction

World Health Organization [1], American Academy of Pediatrics [2], European Society for Pediatric Gastroenterology, Hepatology and Nutrition [3] and Polish Experts Group [4] recommend donor human milk (DHM) as the preferred feeding strategy for preterm infants when the milk of the mother is unavailable. This recommendation is based on conclusive evidence of lower rates of necrotizing enterocolitis (NEC) with the use of DHM feeding compared with preterm infant formulas [1,2,3]. Limited data also suggest that unfortified DHM may be associated with improved feeding tolerance (compared with formula) and with reduced cardiovascular risk factors during adolescence [3]. Schanler et al. [5] reported that only 30% of mothers of extremely preterm infants were able to meet their infants’ needs for feeding throughout the hospitalization of the neonatal intensive care unit (NICU). In the NICU, DHM acts as a bridge until mother’s own milk (MOM) productions meet the infant’s requirements and result in an increased MOM production. In a multicenter study [6], the use of DHM was associated with 10% increase in the rate of MOM utilization and 2.6% decrease in the rate of NEC.

The number of Human Milk Banks in Europe (HMBs) is increasing. Currently, there are 270 active HMB and 15 others are in various stages of planning and development. In Poland, there are 16 HMBs and the first one was opened in 2012 in cooperation with Human Milk Bank Foundation, which provided human milk banking standard, operational procedure and good manufacturing practice of donor milk acquisition and handling. Since there are no consistent European guidelines of HMBs, the consensus statement on established and operation have been published by the European Milk Bank Association. According to those recommendations, the screening process consists of a health questionnaire completed by a potential donor and a personal or telephone interview with the milk bank coordinator who reviews the health questions and assesses the mother’s suitability. Donor selection criteria include serological screening and requirements of healthy lifestyle and dietary habits. In most European HMBs, donors are allowed to donate milk from new-born’s birth, while the term of the end of donation is less specific in HM standard operational procedure [7]. Donors are provided instructions on how to express, store and handle the milk, but with no requirements on how often or how much milk to donate for HMB. Once a woman is accepted as a donor for the HMB, each donated amount of milk is labelled with a unique identification number and milk is stored frozen (−20 °C) in a domestic freezer and subsequently transferred to a milk bank.

**Figure 1:**
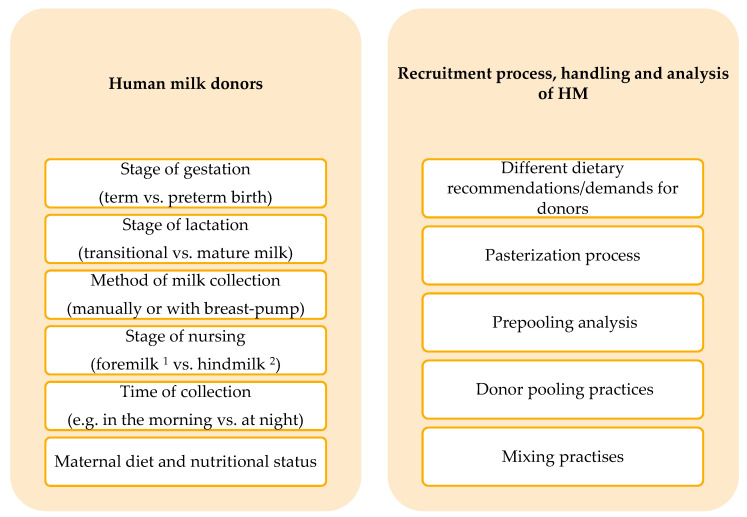
Potential sources of variation in composition of DHM. ^1^ foremilk—milk produced at the beginning of the feeding, ^2^ hindmilk—milk produced at the end of the feeding.

The composition of HM is not only dependent on the individual features of donors but is dynamic in nature, changing over the course of each feeding, over the course of a day, and the course of lactation [8,9,10]. For this reason, HMB routinely pool milk donations from single donors in order to limit extreme variation (Figure 1). Upon pooling, DHM is aliquoted and pasteurized using Holder Pasteurization (62.5 °C for 30 min and rapid cooling). At this stage, bottles are frozen and distributed. It is well documented that Holder Pasteurization destroys cells (e.g., neutrophils and stem cells), reduces the concentration of various bioactive components and affects anti-inflammatory factors of HM. There is very little effect on macro- or micronutrients, including vitamins [2,3]. Additionally, the pattern of milk expression by donors is varied and undefined and the criteria used to select individual donations for pooling are not standardized. Given that, not all findings regarding the composition of HM should be generalized to DHM.

Considering the importance of DHM, we aimed to characterize the variability of energy and macronutrients content in DHM and investigate the impact of maternal factors on feeding practices (exclusive breastfeeding vs. feeding expressed milk) and HM expression pattern on the nutritional value of DHM.

## 2. Materials and Methods

### 2.1. Study Design and Data Collection

This was a retrospective, observational study of the energy and macronutrients composition of HM provided by donors to the HMB in Holy Family Hospital in Warsaw, Poland. Donors registered to the indicated HMB between January 2016 and September 2018 (*n* = 123) included mothers of infants who have been admitted to the NICU and community donors. All registered donors were sent an e-mail containing the information about the research project, however, due to the lack of response or incorrectly completed questionnaires the final analysis was based on 49 participants (response rate was 40%). The questionnaire was conducted by Computer-Assisted Web Interview (CAWI) method and performed within a two-month period. The online questionnaire consisted of questions with two types of responses: close-ended and open-ended. Questions were about maternal anthropometric measurements (height, pre-pregnancy, and current weight), labour and infant-related factors. Information regarding feeding practices and method of milk expression (manually or breast pump) were collected on an ongoing basis, during the milk donation to HMB. Women were also asked about the number of HM expressions for HMB in four time periods (6.00–12.00, 12.00–18.00, 18.00–24.00, 24.00–6:00) in the beginning and in the end of the milk donation to HMB.

The collection of DHM complied with the policies for HM collection established by the European Milk Bank Association (EMBA) and described in the literature [7]. The milk samples that were analyzed (12 mL) were sampled from each pooled batch of an individual’s donated milk prior to pasteurization (from the period of maximum space of two weeks). For each sample, we performed three measurements (3 × 4 mL), and for the result, we used the average of three measurements. For the current work, we analyzed the results at the beginning and at the end of the milk donation to HMB.

### 2.2. Milk Analysis

The HM energy value and macronutrients contents (protein—total/true, fat and carbohydrates) were determined using MIRIS human milk analyzer (HMA) (Miris, Uppsala, Sweden). The HMA uses medium-infrared spectroscopy to detect the levels of macronutrients based on their respective wavelengths as a function of the intensity of the outgoing radiation [11]. The analyzer provided a calculation of energy, using the conversion factors: 4.4, 9.25 and 4.0 kcal per 100 mL for protein, fat, and carbohydrates, respectively. Total protein refers to nitrogen × 6.25, and true protein is the total protein minus 24% for nonprotein nitrogen. Total protein, as reported by the HMA, was converted to bioavailable protein (true protein) for the data analysis using the following equation: total protein (grams) × 0.825. The manufacturer’s protocol was followed according to the user manual, including regular calibration cleaning every ten samples and checking of the machine [12].

### 2.3. Statistical Analysis

Mean, standard deviation, median and inter-quartile range were used to describe quantitative variables. Discrete variables are presented as the number followed by a percentage. Comparison of two groups was performed using *t*-test or paired Wilcoxon test, depending on the distribution of the data. Paired tests were used when applicable. The hypothesis of the normal distribution of the variables was verified using the Shapiro–Wilk test. To evaluate the association between analyzed variables Pearson or Spearman coefficients correlation were computed. The analysis of length of milk donation to HBM was performed in terciles. Thus, analysis of variance and Kruskal–Wallis tests were used for comparisons, χ^2^ test also was used. To establish predictors of longer milk donation to HBM, a logistic regression model was created. The dependent variable in the models was coded as dichotomous using the value of the 2nd tercile of milk donation to HBM length as a threshold. The least absolute shrinkage and selection operator (LASSO) method was used for variable selection and regularization of the model. Results of logistic regression were presented as odds ratio (OR) with a 95% confidence interval (CI) for 1-unit increments of each variable. All computations were performed in R 4.0.5 (R Foundation for Statistical Computing, Vienna, Austria), an environment for statistical programming.

## 3. Results

### 3.1. The Characteristics of Donors and Infants

Mothers and infants’ characteristics are shown in Table 1. Before pregnancy and during the milk donation to HBM, most of the donors had normal weight (*n* = 38, 78%; *n* = 33, 67%, respectively). Before pregnancy, six women were under-weight and five were overweight or obese. The majority of the donors delivered a girl (*n* = 32; 65%) and 35% delivered a boy (*n* = 17). Most of the women were primiparous (73%; *n* = 36). Almost a quarter of the babies (24%, *n* = 12) were born preterm (gestational age < 37 weeks). About one in three mothers (*n* = 18) gave birth by cesarean section, 63% (*n* = 31) of babies were born vaginally.

### 3.2. HM Expression Pattern

In 53% of cases (*n* = 26), women declared exclusive breastfeeding, 37% of babies (*n* = 18) were feeding expressed milk and in 10% (*n* = 5) it was both. The donors were asked about the total number of HM expression for HMB at four different time points: 6.00–12.00, 12.00–18.00, 18.00–24.00, 24.00–6.00. The results are presented in Table 2. We observed statistically significant differences between the number of total daily number of expressions, number and % of afternoon expressions and a number of evenings expressions in the beginning and at the end of milk donation to HMB (V = 388, *p* = 0.006; V = 61, *p* = 0.004; V = 129, *p* = 0.034; V = 57.5, *p* = 0.022, respectively). In the beginning and at the end of milk donation to HMB, the donors most often declared HM expressions at night (39% and 34% of total daily expressions, respectively).

### 3.3. HM Composition in the Beginning and at the End of Milk Donation to HBM

There were 98 donor milk samples, each analyzed in triplicate (*n* = 294 analyses). Table 3 provides results of DHM composition in the beginning and at the end of milk donation to HMB. As presented in Table 1, the mean infants’ age in the beginning of milk donation HMB was 15.4 ± 18.7 weeks, whereas the meantime of milk donation to HMB was 13.2 ± 6.0 weeks. There were no significant differences in macronutrients concentrations in DHM in both time periods, however the energy value and fat concentration were close to being significantly different (*p* = 0.054, *p* = 0.052, respectively).

### 3.4. HM Composition and the Way Infants Feeding

**Figure 2:**
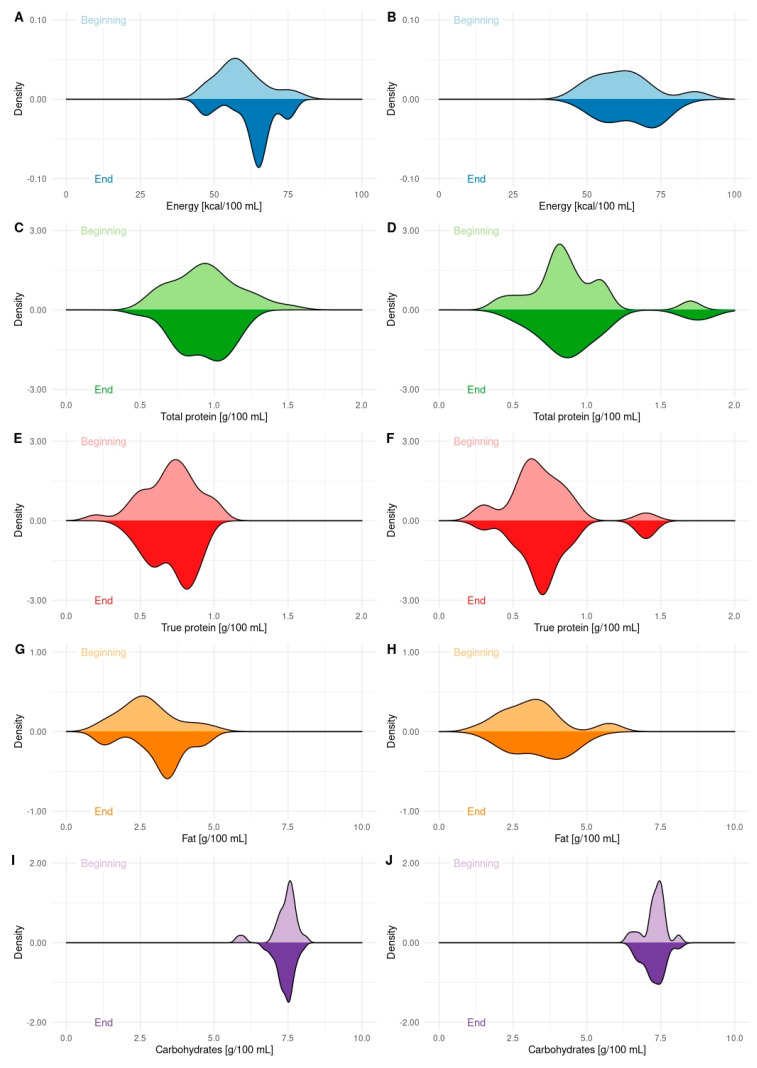
Density plots presenting energy and macronutrients contents of DHM at the beginning (upper part of each panel) and in the end (bottom part of each panel) of the milk donation ratio to HMB, according to the method of infant’s feeding. Panel (**A**,**C**,**E**,**G**,**I**) present the composition of DHM from mothers who exclusively breastfed their infants. Panel (**B**,**D**,**F**,**H**,**J**) present the composition of DHM from mothers who feed their infants only with expressed milk.

HM samples from 18 women who fed their children only with expressed milk were compared with 26 HM samples received from mothers exclusively breastfeeding in the beginning and in the end of milk donation to HBM (Figure 2). The samples from 5 women who fed their children in both ways were not included in the comparison. No differences were found when mothers’ age (30.8 ± 3.0 vs. 30.8 ± 4.6, *p* = 0.974), pre-pregnancy weight (59.1 ± 7.7 vs. 61.3 ± 11.9, *p* = 0.509), pre-pregnancy BMI (20.9 ± 2.2 vs. 22.2 ± 3.6, *p* = 0.189), current weight (60.5 ± 8.4 vs. 62.1 ± 13.8, *p* = 0.658), current BMI (21.4 ± 3.6 vs. 22.4 ± 4.1, *p* = 0.329) were found between these groups. However, higher pregnancy weight gain (15.1 ± 4.8 kg) in mothers exclusively breastfeeding was observed than in mothers who fed their children only with expressed milk (11.3 ± 5.3 kg), *p* = 0.021.

Considering the method of infants feeding, there were also no differences regarding babies’ weight, babies age at the beginning of the milk donation to HMB, or gestational age. No differences were also found according to the feeding pattern (breastfeeding vs. feeding expressed milk) or number of total HM expressions per day, both at the beginning and at the end of milk donation to HBM.

### 3.5. Correlation between Maternal Factors, HM Expression Pattern and DHM Composition

Similarly to Khan et al. [13], we performed an analysis concerning the relationships between the maternal factors, HM expression pattern and DHM composition (Table 4 and Table 5). At the beginning of milk donation to HMB (Table 4), energy and fat content were inversely correlated with donor age (r = −0.364, *p* = 0.01; r = −0.316, *p* = 0.027, respectively). At the end of milk donation to HMB, fat concentration was positively correlated with the percentage of afternoons (12.00–18.00) HM expressions (r = 0.289, *p* = 0.044).

### 3.6. Comparison between Groups Determined Based on Terciles of the Duration of Milk Donation to HMB

Considering the duration of milk donation to HMB, we have determined first and second terciles of this variable. A logistic regression model was built to find factors associated with longer than 16 weeks (2nd tercile) milk donation to HMB. Multiple factors presented in Table 6 were considered. Only mother’s age revealed to be an essential factor according to LASSO analysis (odds ratio 1.15 with 95% confidence interval 0.96–1.42). We observed statistically significant differences in maternal current BMI (0.042) and infants’ age (*p* = 0.026) at the beginning of milk donation to HMB (Table 6).

## 4. Discussion

To the best of our knowledge, our study is the first Polish description of the association between maternal factors and HM expression pattern with DHM composition. We have shown that the expression pattern of milk donated to HMB may influence HM fat concentration. Moreover, we reported that the feeding method (breastfeeding vs. feeding only expressed milk) did not affect energy or macronutrient content in DHM.

HM provides about 68 kcal/100 mL [14,15]. Therefore, it is assumed that an adequate intake for energy requirements in term infants is about this value. Considering that premature infants have higher requirements for growth not accomplished in utero, energy intake from DHM is expected to be higher [14]. In our study, the mean energy contents of DHM samples were 60.6 ± 9.6 kcal/100 mL and 63 ± 9.0 kcal/100 mL at the beginning and at the end of milk donation to HMB, respectively. At the beginning of milk donation to HMB, only 16% of donations had an energy content higher than 68 kcal/100 mL and 27% were lower than 55 kcal/100 mL. At the end of milk donation to HMB, the situation was better; 22% of donations were below 55 kcal/100 mL and 27% had more than 68 kcal/100 mL. Therefore, we can hypothesize that during milk donation to HMB, the energy content may increase, however, the difference had marginal significance. We would like to underline that DHM energy content was similar to the values previously reported in healthy breastfeeding women [16]. Our current results are consistent with Wojcik et al. [17] who reported that 30% of DHM samples had an energy value above 68 kcal/100 mL. We identified and analyzed eight studies [18,19,20,21,22,23,24,25] providing the information of ranges and describing the energy content of DHM, with the mean values between 49.3 kcal/100 mL and 69.3 kcal/100 mL. Seven of them shared a common method of energy content measurement using infrared analysis, as it was in our study. Despite similar average energy content in our study and previous papers, we observed high variability between samples according to energy content. Similar findings were shown by Lamb et al. [18] and Barbarska et al. [22] who reported essential variability in energy content in pre-pasteurized donor milk samples. The results were 48–93 kcal/100 mL and 46–86 kcal/100 mL, respectively, which represents the almost two-fold difference in DHM energy content. These results were consistent with those reported in a systemic review performed by Perrin et. al. [26] and those obtained in our study: 47–87 kcal/100 mL at the beginning of milk donation and 45–81 kcal/100 mL at the end of milk donation to HMB. Despite high variability in DHM energy content, we would like to underline that a similar variability was observed according to HM composition in our previous studies [10,16].

The mean concentrations of fat in our DHM samples were 2.93 ± 1.04 and 3.19 ± 0.98 at the beginning and at the end of milk donation to HMB, respectively. We observed that HM fat content was positively associated with the number of afternoons HM expressions (12.00–18.00). Supporting these findings, Moran-Lev et al. [27] reported that circadian variations in fat content occur consistently over the first seven weeks of lactation in HM expressed by mothers of preterm infants, with evening values 10.9% higher than in the morning samples. Another study [28] of 33 pooled donor milk samples (51 donors) indicated that fat was the most variable HM nutrient, with a coefficient of variation 49%. In this study of randomly pooled donors, the number of donors included in each pool increased the percentage of pools reaching target fat value. Similar results were obtained by Wojcik et al. [16], who reported that in the intra donor analysis, fat content was the most variable (coefficient of variation 21%). Each 1 g/100 mL decrease in the fat content in HM translates into a reduction of 14 kcal/kg/day at feeding volumes of about 160 mL/kg/day [29]. Although in the breastfeeding context this variability is not consequential in terms of the infant’s total daily intake, it influences the nutritional value of pooled DHM. Considering that fat is a major contributor to the energy content in DHM, reducing this variability is justified when feeding the nutritionally at-risk population, which include preterm infants.

The largest study (1119 of HM samples) [26] providing information about the ranges of protein content in DHM reported a minimum total protein concentration of 0.8 g/100 mL and a maximum of 2.2 g/100 mL, which represents an almost three-fold difference. In our study, the ranges were the same in the beginning and at the end of milk donation to HMB and amounted 0.5–1.8 g/100 mL. Similarly to the energy density of our DHM pools, protein concentration in our milk samples was relatively low. As we have mentioned before, standard DHM fortification generally presumes a starting point of 20 kcal/oz (68 kcal/100 mL). Fortifying the average term DHM from this study with a standard commercial HM fortifier concentrate as instructed to reach a presumed 24 kcal/oz (81 kcal/100 mL) would result in a feed consisting of 23.0 kcal/oz (77.8 kcal/100 mL) and 2.38 g/100 mL for protein at the beginning of milk donation to HMB and 23.7 kcal/oz (80.2 kcal/100 mL), 2.38 g/100 mL for protein, at the end of milk donation to HMB. In the study performed by Young et al. [30] obtained values were lower and amounted 21.5 kcal/oz and 1.96 g/100 mL. Another report [26] indicated that 75% of mature DHM samples would not meet protein targets after standard fortification practices without exceeding intakes of 160 mL/kg/day. Therefore, these results need to be considered when providing DHM to low birth-weight and premature infants. We did not observe any correlations between maternal factors, feeding practices (breastfeeding vs. feeding expressed milk) and DHM composition. In other studies, investigating associations between HM protein concentration and maternal nutritional status, the results were contradictory. Some authors [9,31] indicated that maternal BMI was positively correlated with protein content. However, it must be stressed that the participants of this study were not donors of HM, and that BMI is not a direct measure of maternal adiposity, so that the strength of the relationship between protein concentration and BMI may not reflect the true value of these associations.

Considering the feeding method, we did not observe any differences in energy and macronutrients content of DHM, which is particularly important in feeding preterm new-borns who cannot be breastfed by their mothers, as well as other infants fed with expressed milk (e.g., infants whose mothers return to work or school). There are a variety of indications for HM expression and the type and approach to milk expression may vary by particular indication. Most often, mothers of premature or critically ill babies are forced to initiate lactation by pumping because the babies are unable to suck. This may also be necessary for new-borns with a cleft palate, who may not be able to generate adequate negative pressure to feed directly from their mothers’ breast [32]. Some women have infants who have difficulty establishing an effective latch, or mothers do not want to breastfeed directly. These mothers are usually strongly motivated to feed their babies with expressed milk, even though, it is difficult to maintain lactation through pumping alone. Geraghty et al. [33] reported that among 80 mothers of healthy term infants who fed their babies only with expressed milk, none were able to provide breast milk through six months. Concerning these difficulties, Wu et al. [34] performed a study which aimed to improve the milk expression in mothers of the preterm infants by the recording breast milk pumping diaries. The developed diary contained all the important information of pumping, including the date, time, and milk volume of each pumping. The total amount of milk pumped within that day and the total milk feeding volume were in the recorded diary. Accordingly, the difference of milk amount between pumping and feeding was then calculated. The authors found that in eight months, the ratio of expressed HM delivered to the NICU rose significantly to 53.3% (*p* < 0.01). Both the ratios of breastfeeding and mixed feeding also rose to 23.8% and 55.3%, respectively. The composition and volume of expressed milk at the initial stage of lactation is highly dependent on breast emptying, the number of pumping sessions, and the length of each session [35,36]. To our knowledge, our results show for the first time that the composition of expressed milk in the later stages of lactation is comparable to mothers’ own milk when breastfeeding. We believe that these findings may be supportive for pumping-dependent mothers.

The dataset represents the composition of mature milk, primarily donated by women who delivered at term. This study is limited because all milk samples came from Polish donors, so they may not necessarily be reflective of donor populations of HMBs serving specific communities. The sample size was relatively small, however, in our opinion, information coming from donors, especially those concerning HM expression pattern, are unique. Most researchers [18,37,38,39] using mid-infrared transmission spectroscopy to assess the nutritional value of DHM, did not observe significant differences in macronutrient content between the pre- and post-pasteurization process (Holder 62.5 °C). Lamb et al. [18] reported that macronutrient deficiencies in pre- and post-pasteurization DHM samples were within the coefficient variation of the machine (15% protein, 12% fat and 15% carbohydrates) [12]. Therefore, our pre-pasteurization analyses are comparable to those after Holder pasteurization.

Knowledge of factors that influence the composition of DHM and the routine analysis of expressed and donated milk have the potential to match an infant’s current energy and macronutrients requirements, which may reduce under- or over-fortification. Some researchers [40,41] have reported success with individualized fortification regarding weight and growth improvements with milk fortification after HM analysis. Future research for the DHM should include a further cross-sectional observational study with the collection of detailed donor information and characteristics of expression and feeding practices to further evaluate the pooling processes and the effect on HM composition.

Donor selection for HMBs is essential, both to ensure the safety and nutritional quality of the DHM, as well as to ensure that the future donor and her breastfeeding infant do not come to harm through the donation experience. In regard to results of our study, the development of guidelines and selection criteria for HM donors may influence DHM composition. Donor selection criteria should be more detailed and, except for currently valid recommendations, pattern of milk expression could be involved. Therefore, there is a window of opportunity to optimize the nutrition strategy for infants who are fed with DHM.

## 5. Conclusions

We did not observe any significant differences in HM composition donated by mothers who exclusively breastfed their infants and those who fed expressed milk. However, we observed that HM expression patterns, such as the number of expressions at a different time of a day, may influence the fat concentration in HM. Owing to the fact that the present study has the limitation of small sample size, further research on a large scale, involving different populations to investigate factors connected with donors that are associated with DHM composition, are warranted. Hence, recruiting HM donors with a desirable profile and pattern of milk expression may contribute to the increase in the nutritional value of DHM, while reducing the need for HM fortifiers.

## Figures and Tables

**Table 1:** The descriptive characteristics of the mothers and infants.

Parameter.	Mean ± SD	Median (IQR ^1^)
**Mothers**		
Age (years)	30.7 ± 3.6	30.5 (29.1–32.8)
Pre-pregnancy weight (kg)	59.7 ± 9.9	58.0 (53.0–64.0)
Pre-pregnancy BMI (kg/m^2^)	21.4 ± 2.9	20.5 (19.5–22.8)
Pregnancy weight gain (kg)	13.4 ± 5.6	14.0 (9.0–17.0)
Current weight (kg)	61.0 ± 11.1	60.0 (53.0–65.0)
Current BMI (kg/m^2^)	21.9 ± 3.4	21.5 (19.5–23.7)
Time of milk donation to HMB (weeks)	13.2 ± 6.0	14.0 (8.0–17.0)
**Infants**		
Week of delivery	37.8 ± 3.7	39.0 (37.0–40.0)
Birth weight (g)	3117 ± 777	3300 (2900–3650)
Infants’ age in the beginning of milk donation to HMB (weeks)	15.4 ± 18.7	10.0 (5.0–18.0)

^1^ IQR, interquartile range.

**Table 2:** The number and percentage ^1^ of HM expressions at different time points.

	The Beginning of Milk Donation to HBM		The End of Milk Donation to HBM		*p*-Value ^2^
Parameter	Mean ± SD	Median (IQR ^3^)	Mean ± SD	Median (IQR ^3^)	
Number of total daily HM ^4^ expressions	8.2 ± 3.8	9.0 (5–11)	9.5 ± 3.6	10 (8–12)	0.006 *
Number of mornings HM expressions (6.00–12.00)	1.9 ± 1.4	2 (1–3)	2.1 ± 1.3	2 (1–3)	0.212
% of mornings HM expressions (6.00–12.00)	21 ± 13	25 (11–31)	22 ± 12	25 (14–29)	0.784
Number of afternoons HM expressions (12.00–18.00)	1.9 ± 1.5	2 (1–3)	2.3 ± 1.4	3 (1–4)	0.004 *
% of afternoons HM expressions (12.00–18.00)	21 ± 15	25 (9–31)	23 ± 13	25 (14–31)	0.034 *
Number of evenings HM expressions (18.00–24.00)	1.8 ± 1.3	2 (1–3)	2.2 ± 1.3	2 (1–3)	0.022 *
% of evenings HM expressions (18.00–24.00)	19 ± 12	22 (14–30)	21 ± 11	22 (14–30)	0.139
Number of nights HM expressions (24.00–6.00)	2.6 ± 1.3	3 (1–4)	2.9 ± 1.2	3 (2–4)	0.069
% of nights HM expressions (24.00–6.00)	39 ± 24	33 (25–44)	34 ± 18	31 (25–40)	0.178

^1^ The percentage of HM expressions at different time points was calculated as the ratio between the number of HM expressions at each time point and the number of total daily HM expressions, then multiplied by 100%. ^2^ Wilcoxon signed rank test for paired samples. ^3^ IQR, interquartile range. ^4^ HM–human milk. * *p* < 0.05.

**Table 3:** Comparison of DHM composition in the beginning and at the end of milk donation to HMB.

	The Beginning of Milk Donation to HBM		The End of Milk Donation to HBM		*p*-Value ^1^
	Mean ± SD	Median (IQR ^2^) Min-Max	Mean ± SD	Median (IQR ^2^) Min-Max	
Energy (kcal/100 mL)	60.6 ± 9.6	59 (54–66) 47.4–86	63.0 ± 9.0	64 (57–69) 45–81	0.054
Total protein (g/100 mL)	0.94 ± 0.26	0.9 (0.8–1.1) 0.4–1.7	0.94 ± 0.24	0.9 (0.8–1.1) 0.5–1.8	0.819
True protein (g/100 mL)	0.72 ± 0.22	0.7 (0.6–0.8) 0.3–1.4	0.73 ± 0.20	0.7 (0.6–0.8) 0.3–1.4	0.897
Fat (g/100 mL)	2.93 ± 1.04	2.8 (2.2–3.5) 1.1–5.9	3.19 ± 0.98	3.4 (2.5–2.8) 1.1–5.1	0.052
Carbohydrates (g/100 mL)	7.35 ± 0.44	7.5 (7.2–7.6) 5.8–8.1	7.35 ± 0.34	7.4 (7.2–7.6) 6.6–8.1	0.856

^1^ Wilcoxon signed-rank test for paired samples. ^2^ IQR, interquartile range.

**Table 4:** Correlations ^1^ between maternal factors, HM expression pattern and DHM composition at the beginning of milk donation to HMB.

Parameter	Correlation Coefficients				
	Energy	Total Protein	True Protein	Fat	Carbohydrates
**Maternal factors**					
Age (years)	−0.364 *	0.073	0.072	−0.316 *	0.064
Pre-pregnancy BMI (kg/m^2^)	0.014	−0.141	−0.168	0.073	−0.128
Pregnancy weight gain (kg) ^1^	−0.264	−0.019	−0.043	−0.211	−0.037
Current BMI (kg/m^2^)	−0.089	−0.095	−0.143	−0.041	−0.165
**HM expression pattern**					
Number of total daily HM expressions	−0.167	−0.232	−0.232	−0.183	0.172
					
% of mornings HM expressions (6.00–12.00)	−0.269	−0.033	−0.026	−0.264	0.109
% of afternoons HM expressions (12.00–18.00)	−0.182	−0.31 *	−0.227	−0.183	0.042
% of evenings HM expressions (18.00–24.00)	−0.052	−0.264	−0.253	−0.015	0.006
% of nights HM expressions (24.00–6.00)	0.25	0.265	0.168	0.251	−0.133

^1^ Depending on the distribution of variables, Pearson or Spearman correlation coefficients were used. * *p* < 0.05.

**Table 5:** Correlations ^1^ between maternal factors, HM expression pattern and DHM composition in the end of milk donation to HMB.

Parameter	Correlation Coefficient				
	Energy	Total Protein	True Protein	Fat	Carbohydrates
**Maternal factors**					
Age (years)	0.158	0.023	0.051	0.153	0.033
Pre-pregnancy BMI (kg/m^2^)	−0.065	0.001	0.013	−0.056	−0.204
Pregnancy weight gain (kg)	−0.189	−0.174	−0.21	−0.158	0.006
Current BMI (kg/m^2^)	−0.052	−0.074	−0.097	−0.06	−0.154
**HM expression pattern**					
Number of total daily HM expressions	−0.034	−0.073	−0.135	−0.022	0.169
% of mornings HM expressions (6.00–12.00)	0.131	0.211	0.153	0.083	0.132
% of afternoons HM expressions (12.00–18.00)	0.235	−0.058	−0.074	0.289 *	−0.158
% of evenings HM expressions (18.00–24.00)	0.038	−0.102	−0.014	0.067	−0.056
% of nights HM expressions (24.00–6.00)	−0.246	−0.154	−0.122	−0.265	0.159

^1^ Depending on the distribution of variables, Pearson or Spearman correlation coefficients were used. * *p* < 0.05.

**Table 6:** Comparison between groups ^1^ selected based on terciles of the duration of milk donation to HMB.

	Group 1		Group 2		Group 3		*p*-Value
	Mean ± SD	Median (IQR ^2^)	Mean ± SD	Median (IQR ^2^)	Mean ± SD	Median (IQR ^2^)	
Age (years)	30.2 ± 3.8	30.5 (28.1–32.0)	30.7 ± 2.9	31.5 (29.7–32.6)	31.1 ± 3.9	30.1 (29.3–33.3)	0.762 ^3^
Pregnancy weight gain (kg)	12.6 ± 6.4	12.0 (8.0–16.5)	14.0 ± 5.3	15.0 (10.5–16.8)	13.6 ± 5.3	12.8 (9.8–16.2)	0.794 ^3^
Pre-pregnancy BMI (kg/m^2^)	21.0 ± 2.3	20.4 (19.6–22.5)	22.6 ± 4.2	21.8 (20.2–24.2)	21.0 ± 2.1	20.4 (19.3–22.6)	0.218 ^3^
Current BMI (kg/m^2^)	21.7 ± 3.0	21.5 (19.6–23.2)	23.7 ± 4.4	23.5 (20.2–25.4)	20.8 ± 2.3	21.0 (18.5–22.1)	0.042 ^3,^*
Week of delivery	36.8 ± 4.5	39.0 (37.0–39.5)	38.1 ± 3.5	39.5 (38.0–40.0)	38.4 ± 3.0	39.0 (37.0–40.0)	0.548 ^4^
Infant’s birth weight (g)	2936.7 ± 1012.0	3280.0 (2887.0–3550.0)	3277.0 ± 729.0	3510.0 (3239.0–3650.0)	3140.0 ± 602.0	3125.0 (2788.0–3564.0)	0.432 ^4^
Infants’ age at the beginning of milk donation to HMB (weeks)	14.0 ± 28.9	5 (3–12)	20.9 ± 16.5	17.0 (7.5–29.5)	12.7 ± 7.0	10.0 (7.8–17.2)	0.026 ^4,^*
Time of milk donation to HMB (weeks)	5.8 ± 2.4	7.0 (3.0–7.0)	13.2 ± 1.5	13.5 (12.2–14.0)	18.7 ± 3.0	17.0 (16.0–22.0)	<0.001 ^3,^*

^1^ Group 1—Milk donation HBM < 11 weeks, Group 2—Milk donation to HMB between 11 and 15 weeks, Group 3—Milk donation to HMB ≥ 16 weeks. ^2^ IQR, interquartile range. ^3^ One-way ANOVA on ranks. ^4^ Kruskal-Wallis test by ranks. * *p* < 0.05.

## Data Availability

The data presented in this study are available on request from the corresponding author. The data are not publicly available due to information that could compromise the privacy of research participants.
